# Human phenotype ontology annotation and cluster analysis for pulmonary atresia to unravel clinical outcomes

**DOI:** 10.3389/fcvm.2022.898289

**Published:** 2022-07-29

**Authors:** Bingyan Shu, Huayan Shen, Xinyang Shao, Fengming Luo, Tianjiao Li, Zhou Zhou

**Affiliations:** Fuwai Hospital, Chinese Academy of Medical Sciences and Peking Union Medical College, Beijing, China

**Keywords:** pulmonary atresia, Human Phenotype Ontology, unsupervised cluster analysis, Kaplan-Meier curves, Cox proportional hazards regression

## Abstract

**Background:**

Pulmonary atresia (PA) is a heterogeneous congenital heart defect and ventricular septal defect (VSD) is the most vital factor for the conventional classification of PA patients. The simple dichotomy could not fully describe the cardiac morphologies and pathophysiology in such a complex disease. We utilized the Human Phenotype Ontology (HPO) database to explore the phenotypic patterns of PA and the phenotypic influence on prognosis.

**Methods:**

We recruited 786 patients with diagnoses of PA between 2008 and 2016 at Fuwai Hospital. According to cardiovascular phenotypes of patients, we retrieved 52 HPO terms for further analyses. The patients were classified into three clusters based on unsupervised hierarchical clustering. We used Kaplan–Meier curves to estimate survival, the log-rank test to compare survival between clusters, and univariate and multivariate Cox proportional hazards regression modeling to investigate potential risk factors.

**Results:**

According to HPO term distribution, we observed significant differences of morphological abnormalities in 3 clusters. We defined cluster 1 as being associated with Tetralogy of Fallot (TOF), VSD, right ventricular hypertrophy (RVH), and aortopulmonary collateral arteries (ACA). ACA was not included in the cluster classification because it was not an HPO term. Cluster 2 was associated with hypoplastic right heart (HRH), atrial septal defect (ASD) and tricuspid disease as the main morphological abnormalities. Cluster 3 presented higher frequency of single ventricle (SV), dextrocardia, and common atrium (CA). The mortality rate in cluster 1 was significantly lower than the rates in cluster 2 and 3 (*p* = 0.04). Multivariable analysis revealed that abnormal atrioventricular connection (AAC, *p* = 0.011) and persistent left superior vena cava (LSVC, *p* = 0.003) were associated with an increased risk of mortality.

**Conclusions:**

Our study reported a large cohort with clinical phenotypic, surgical strategy and long time follow-up. In addition, we provided a precise classification and successfully risk stratification for patients with PA.

## Introduction

Pulmonary atresia is a rare but heterogeneous congenital heart defect defined as the absence of direct communication between the ventricular and the pulmonary vascular bed (1). Currently, the treatment method for PA always depends on the operation, which optimizes the chance of biventricular circulation by establishing flow from the right ventricle to the pulmonary system (1, 2). In general, multiple surgical treatments are inevitable for diagnosed patients to avoid high early mortality (3, 4). Even though patients survive PA, they may still have a high possibility of short-term and long-term complications, including heart failure, respiratory failure, pulmonary infection and death (5, 6). Moreover, the costs of treatment impose a heavy financial burden on families and society (7).

Treatment and prognosis depend largely on the cardiac morphology (2). Ventricular septal defect is the most vital factor for the conventional classification of PA patients, PA-VSD allows blood to flow into and out of the right ventricle and helps the ventricle develop (2). However, this simple dichotomy could not fully describe the cardiac morphologies and pathophysiology in such a complex and heterogeneous congenital heart defect. Montanaro et al. added the third group: PA-VSD with complex univentricular anatomy (2). PA patients generally have diverse concomitant cardiac malformations, such as VSD, ASD, RVH, tricuspid valve disease, and abnormal aortic morphology (AAM) (8, 9). Consequently, we need a more elaborate classification of PA patients to help clinical decision-making and prognosis analysis.

In this study, we utilized the Human Phenotype Ontology, a database that provides a comprehensive logical standard to describe and computationally analyze phenotypic abnormalities found in human disease (10), to explore the phenotypic patterns of PA and the phenotypic influence on prognosis. We aimed to draw a comprehensive cardiovascular phenotype profile for PA based on an enormous cohort, stratify patients with unsupervised clustering analysis based on HPO, and provide novel clinical implications and prognostic information.

## Materials and methods

### Patients and information collection

We searched for patients with diagnoses of PA at Fuwai Hospital between 2008 and 2016 from the electronic medical records (EMR) system. The patients underwent echocardiography and electrocardiography and received surgical treatment at Fuwai Hospital. A total of 786 patients were recruited, and the data collection was completed by two medical students, which was further confirmed by a specialized cardiologist. The data included demographic information (age, sex, BMI, family history, etc.), clinical history (echocardiogram, electrocardiograph, diagnosis), surgical details (surgical diagnosis, surgical history, complete repair or shunts), and revisit records (echocardiogram, electrocardiograph). Patients who were older than 18 years old of surgery or had incomplete records, no surgical treatment in Fuwai Hospital, or severe extracardiac disease were excluded. Eventually, a total of 715 patients were enrolled for further analyses. The requirement to obtain informed consent was waived because of the retrospective nature of the present study. The study was approved by the Institutional Review Board of the Fuwai Hospital (2017-877).

### Annotation of cardiac phenotypes by human phenotype ontology

All cardiovascular phenotypes were extracted from echocardiograms, electrocardiographs, admitting diagnoses, surgical diagnoses, and discharge diagnosis, and annotated to the standard HPO term online.^1^ A total of 52 HPO terms were retrieved for further analyses, and the details are summarized in Supplementary Table 1.

The Human Phenotype Ontology as a tool for annotating and analyzing human hereditary disease has been defined (11–14). The definition of information content (IC) for the HPO terms was previous reported (13). The *p*_*t*_ is the frequency of the term in the PA patients. The IC of term *t* is given:


$$\text{IC}\,\left(t\right)=-l\,o\,g\,p_{t}$$

The next step of this metric was applied to measure the similarity for two terms in an ontology. A term in one case of HPO might have multiple parent terms, and thus, a pair of terms might have more than one path of common ancestors. Denoting the set of all common-ancestor terms of terms *s* and *t*, the similarity between two terms, *s* and *t*, is defined as:


$$\text{sim}\,\left(s,t\right)=\begin{array}{c} \max\\ v\in a\,n\,c\,\left(s\right)\,a\,n\,c\,\left(t\right) \end{array}\,I\,C\,\left(v\right)$$

The anc(x) denotes the ancestor terms of x.

The patients who were included have at least one HPO term, and the next step of clustering was calculating the similarity matrix (sim_mat) in pairwise patients (*D_*a*_, D_*b*_*) based on “between term set” similarities by the following equation:


$$s\,i\,m\,\left(D_{a},D_{b}\right)=\frac{1}{2\,\left|D_{a}\right|}\,\sum_{s\in D_{a}}\begin{array}{c} \max s\,i\,m\,\left(s,t\right)\\ t\in D_{b} \end{array}+\frac{1}{2\,\left|D_{b}\right|}\,\sum_{s\in D_{b}}\begin{array}{c} \max s\,i\,m\,\left(s,t\right)\\ t\in D_{a} \end{array}$$

We calculated a distance matrix (max(sim_mat) – sim_mat) based on a similarity matrix (sim_mat, the similarity in any two of the patients) guided by the R package “ontologySimilarity.”^2^ Then, we used the R package “pheatmap” to perform unsupervised hierarchical clustering according to the distance matrix. The complete linkage method was selected for hierarchical clustering by default, and the parameter in the function “pheatmap” was employed. The distance between two clusters is the maximal distance between any two elements in each cluster. The parameter “cutree_col” was set to three to acquire three phenotypically heterogeneous clusters.

All analyses were conducted using the packages “ontologySimilarity,” “ontologyIndex,” and “ontologyPlot” in R.

### Follow-up and mortality data

We conducted a follow-up telephone interview for all included patients to inquire about their postoperative situation, including survival, reoperation, revisit records in other hospitals, family history of congenital heart disease. A total of 366 patients were reached, and the rate of loss to follow-up was 48.81%. For patients who could not be interviewed, we regarded the last revisit records in our hospital as the outcome. Finally, we excluded 63 patients who were lost to follow-up and had no revisit records, accounting for 8.9% of the enrolled patients. There were no significant differences between the excluded patients and the 648 patients included for final analysis. All-cause mortality was defined as the endpoint.

### Statistical analysis

A *p*-value < 0.05 was considered statistically significant. All analyses were performed using R Software V.4.1.1 and SPSS V.22.0.

The clinical characteristics and phenotypes are summarized as the mean ± SD for continuous variables or frequencies and percentages for categorical variables. Comparisons of the differences between clusters of continuous variables were studied using the ANOVA in the situation of normal distribution, using the non-parametric Wilcoxon rank sum test for skew distribution. The frequency was compared across levels of each explanatory variable using the Pearson *χ^2^* test or Fisher’s exact test when group size was less than 10 for categoric variables.

Survival was estimated using Kaplan–Meier curves and the log-rank test to compare survival between clusters. The survival time of the included patients was set at definitive surgery and ended at endpoints (death, the last revisit or the last follow-up).

Univariate Cox proportional hazards regression modeling was used to investigate potential risk factors for clinical adverse outcomes. We included the HPO-based clusters, the HPO terms with a percentage greater than 5%, different types of surgery, and demographics, including age, sex, body-mass index (BMI) and familial history, as potential prognostic markers to test in the univariate analysis model. For the multivariate Cox proportional hazards regression model, we used the variables that were significant in the univariate Cox model. Other variables included the HPO-based clusters, demographic information, different types of surgery, and the HPO terms, which had a percentage greater than 25%.

## Results

### Patient characteristics

**FIGURE 1:**
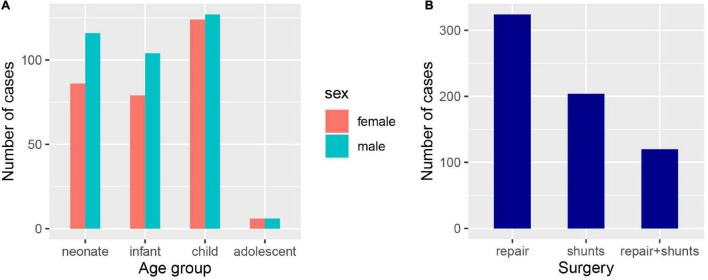
Clinical characteristics of the confirmed PA patients. **(A)** The number of PA patients in different age and gender groups, neonate (age < 1), infant (1 ≤ age ≤ 2), child (3 ≤ age ≤ 12), and adolescent (13 ≤ age < 18). **(B)** The number of PA patients undergone different types of surgery.

According to the inclusion and exclusion criteria, we identified 648 patients with hospitalizations that were included in the study. The median age of this cohort was 2 years, and patients in cluster 3 were significantly older than those in cluster 1 and 2 (*p* < 0.001). We classified all patients into four age groups, neonate, infant, child, and adolescent (Figure 1A). There were 295 (45.55%) female patients in the cohort, and the proportion of females in cluster 3 (25%) was lower than that in cluster 1 (46.17%) and cluster 2 (48.93%) (*p* = 0.048). Regarding the type of procedure during hospitalization, cluster 1 (53.45%) had more patients undergoing repair than the other two clusters (37.23%, 31.25%) (*p* = 0.002), and the use of shunts or was not different among the three clusters (Figure 1B). The baseline characteristics of the entire cohort are summarized in Table 1.

**TABLE 1:** Clinical characteristics of the PA patients.

Variables	All cohort (*n* = 648)	Cluster 1 (*n* = 522)	Cluster 2 (*n* = 94)	Cluster 3 (*n* = 32)	*P*-value
Age at definitive surgery, y	2 (0.75–5)	2 (0.83–4.75)	0.71 (0.25–4)	5.5 (3–8.25)	< 0.001*
Age at follow- up, m	5.33 (1.5–8.33)	5.42 (1.6–8.33)	5.04 (1.83–7.52)	6 (0.5–8.08)	0.286
Female	295 (45.52)	241 (46.17)	46 (48.93)	8 (25)	0.048*
BMI	15.82 ± 12.98	15.85 ± 14.31	15.39 ± 5.83	16.53 ± 11.46	0.954
**Surgical strategy**					
Repair	324 (50)	279 (53.45)	35 (37.23)	10 (31.25)	0.002*
Shunts	204 (31.48)	150 (28.74)	38 (40.43)	16 (50)	0.119
Repair+Shunts	120 (18.52)	93 (17.82)	21 (22.34)	6 (18.75)	0.516
**Associated anomalies**					
TOF	122 (18.83)	116 (22.22)	1 (1.06)	5 (15.63)	< 0.001*
VSD	512 (79.01)	482 (92.34)	9 (9.57)	21 (65.63)	< 0.001*
ASD	224 (34.57)	161 (30.84)	54 (57.45)	9 (28.13)	< 0.001*
PDA	490 (75.62)	392 (75.1)	89 (94.68)	9 (28.13)	< 0.001*
PFO	211 (32.56)	167 (31.99)	44 (46.81)	0 (0)	< 0.001*
RVH	203 (31.33)	186 (35.63)	9 (9.57)	8 (25)	< 0.001*
ACA	281 (43.36)	267 (51.15)	1 (1.06)	13 (40.63)	< 0.001*
HRH	44 (6.79)	7 (1.34)	35 (37.23)	2 (6.25)	< 0.001*
SV	45 (6.94)	30 (5.75)	8 (8.51)	7 (21.88)	0.005*
Dextrocardia	44 (6.79)	29 (5.56)	8 (8.51)	7 (21.88)	0.004*
CA	25 (3.86)	11 (2.11)	6 (6.38)	8 (25)	< 0.001*
LSVC	93 (14.35)	82 (15.71)	3 (3.19)	8 (25)	< 0.001*
**Pulmonary artery abnormality**					
PAH1	6 (0.93)	5 (0.96)	1 (1.06)	0 (0)	1
PAH2	6 (0.93)	5 (0.96)	0 (0)	1 (3.13)	0.299
PAS	49 (7.56)	33 (6.32)	14 (14.89)	1 (3.125)	0.019*
**Aortic abnormality**					
RAA	49 (7.56)	45 (8.62)	0 (0)	4 (12.5)	< 0.001*
DAA	3 (0.46)	3 (0.57)	0 (0)	0 (0)	1
**Aortic valve abnormality**					
AR/AI	16 (2.47)	14 (2.68)	2 (2.13)	0 (0)	1
AVS	1 (0.15)	1 (0.19)	0 (0)	0 (0)	1
BAV	3 (0.46)	2 (0.38)	1 (1.06)	0 (0)	0.478
**Tricuspid valve abnormality**					
TR/TI	99 (15.28)	33 (6.32)	64 (68.09)	2 (6.25)	< 0.001*
DTV	17 (2.62)	2 (0.38)	15 (15.96)	0 (0)	< 0.001*
TS	19 (2.93)	4 (0.77)	15 (15.96)	0 (0)	< 0.001*
TA	18 (2.78)	2 (0.38)	12 (12.77)	4 (12.5)	< 0.001*
EATV	2 (0.31)	1 (0.19)	1 (1.06)	0 (0)	0.351
**Mitral valve abnormality**					
MR/MI	16 (2.47)	11 (2.11)	5 (5.32)	0 (0)	0.135
MA	3 (0.46)	1 (0.19)	1 (1.06)	1 (3.13)	0.048*
**Other**					
DORV	20 (3.09)	17 (3.26)	1 (1.06)	2 (6.25)	0.207
RVD	6 (0.93)	6 (1.19)	0 (0)	0 (0)	0.704
RVOTO	6 (0.93)	5 (0.96)	0 (0)	1 (3.13)	0.299
HLH	5 (0.77)	2 (0.38)	1 (1.06)	2 (6.25)	0.013*
LVH	3 (0.46)	1 (0.19)	1 (1.06)	1 (3.13)	0.048*
RAE	8 (1.23)	4 (0.77)	3 (3.19)	1 (3.13)	0.052
Mesocardia	11 (1.7)	9 (1.72)	1 (1.06)	1 (3.13)	0.65
ASOCS	23 (3.55)	18 (3.45)	3 (3.19)	2 (6.25)	0.567
ACD/ECD	22 (3.4)	15 (2.87)	4 (4.26)	3 (9.38)	0.118
ACAM1	6 (0.93)	2 (0.38)	3 (3.19)	1 (3.13)	0.016*
AAC	20 (3.09)	15 (2.87)	1 (1.06)	4 (12.5)	0.015*
AAVM	20 (3.09)	13 (2.49)	5 (5.32)	2 (6.25)	0.144
PI	3 (0.46)	1 (0.19)	2 (2.13)	0 (0)	0.098
APV	15 (2.31)	8 (1.53)	5 (5.32)	2 (6.25)	0.018*
TGA	75 (11.57)	64 (12.26)	7 (7.45)	4 (12.5)	0.425
ACAM2	20 (3.09)	14 (2.68)	3 (3.19)	3 (9.36)	0.099
ATW	27 (4.17)	12 (2.3)	6 (6.38)	9 (28.13)	< 0.001*
ASTS	14 (2.16)	6 (1.15)	5 (5.32)	3 (9.38)	0.001*
RBBB	20 (3.09)	11 (2.11)	3 (3.19)	6 (18.75)	< 0.001*
PVC/VPB	1 (0.15)	0 (0)	1 (1.06)	0 (0)	0.194

Data are given as mean ± SD, median (25th– 75th percentiles), or n (%). *P-value < 0.05 was considered statistically significant. TOF, tetralogy of Fallot; VSD, ventricular septal defect; ASD, atrial septal defect; PDA, patent ductus arteriosus; POF, patent foramen ovale; RVH, right ventricular hypertrophy; ACA, aortopulmonary collateral arteries; HRH, hypoplastic right heart; SV, single ventricle; CA, common atrium; LSVC, persistent left superior vena cava; PAH1, pulmonary arterial hypertension; PAH2, pulmonary artery hypoplasia; PAS, pulmonary artery stenosis; RAA, right aortic arch; DAA, double aortic arch; AR/AI, aortic regurgitation/aortic insufficiency; AVS, aortic valve stenosis; BAV, bicuspid aortic valve; TR/TI, tricuspid regurgitation/tricuspid insufficiency; DTV, dysplastic tricuspid valve; TS, tricuspid stenosis; TA, tricuspid atresia; EATV, Ebstein anomaly of the tricuspid valve; MR/MI, mitral regurgitation/mitral insufficiency; MA, mitral atresia; DORV, double outlet right ventricle; RVD, right ventricular dilatation; RVOTO, right ventricular outflow tract obstruction; HLH, hypoplastic left heart; LVH, Left ventricular hypertrophy; RVE, right atrial enlargement; ASOCS, abnormal spatial orientation of the cardiac segments; ACD/ECD, atrioventricular canal defect/Endocardial cushion defect; ACAM1, abnormal cardiac atrium morphology; AAC, abnormal atrioventricular connection; AAVM, abnormal atrioventricular valve morphology; PI, pulmonary insufficiency; APV, Abnormality of the pulmonary veins; TGA, transposition of the great arteries; ACAM2, abnormal coronary artery morphology; ATW, Abnormal T-wave; ASTS, Abnormal ST segment; RBBB, right bundle branch block; PVC/VPB, premature ventricular contraction/ventricular premature beat.

### Standardization and annotation for phenotypes

**FIGURE 2:**
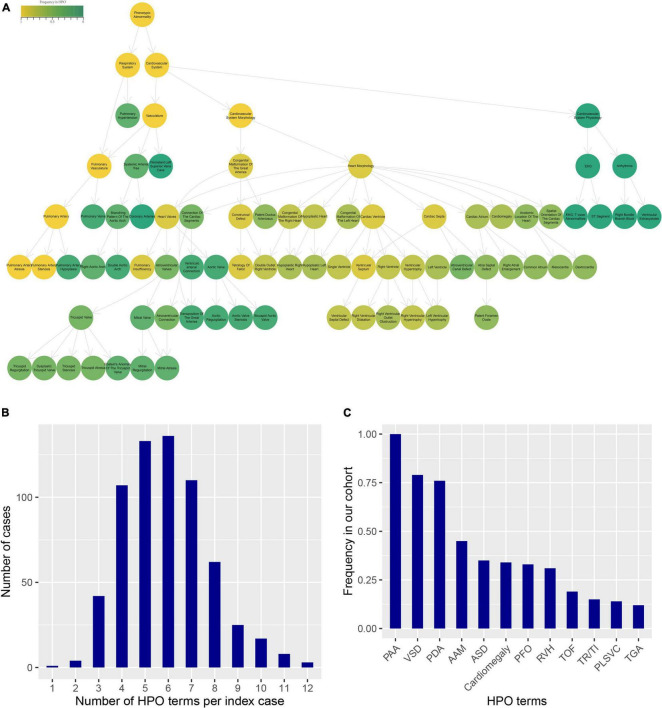
HPO terms annotated for the clinical phenotype of PA patients. **(A)** The ontology plot presented the relationship of all HPO terms. The circles with border showed the all phenotypes in PA patients. The color indicated the frequency of terms in the HPO database. The arrows were “is-a” relations between terms in the ontology. **(B)** The distribution of the number of HPO terms per index case. **(C)** The frequency distribution of HPO terms in our cohort.

We obtained the patients’ clinical records and extracted the diagnostic terms in Chinese EMRs. If the diagnostic term could be matched to the HPO database, but not supported in the R package “OntologySimilarity,” we select its superior term instead. According to the process, a total of 52 HPO terms in cardiovascular disease-related diagnostics were annotated for further analyses, and detailed information was enumerated in Supplementary Table 1. The Figure 2A shows an ontology plot to display the categories and relationships of phenotype terms. According to the ontology plot, the main types of PA-associated phenotypes were morphology. In this cohort, three patients had a maximum of twelve HPO terms, and just one presented a minimum of one term. The majority of patients had four to seven HPO terms, with a median number of six terms annotated to each patient (Figure 2B). The frequency distribution of all HPO terms is presented in Figure 2C. VSD and patent ductus arteriosus (PDA) were the most common phenotypes, followed by AAM, ASD, cardiomegaly, patent foramen ovale (PFO) and RVH. We performed unsupervised clustering of the HPO-encoded phenotype data in order to obtain an undirected characterization of different subgroups within the heterogeneous BPD collection and assess whether particular sets of HPO terms tended to co-occur among cases in these groups.

### Human phenotype ontology clustering for pulmonary atresia patients

**FIGURE 3:**
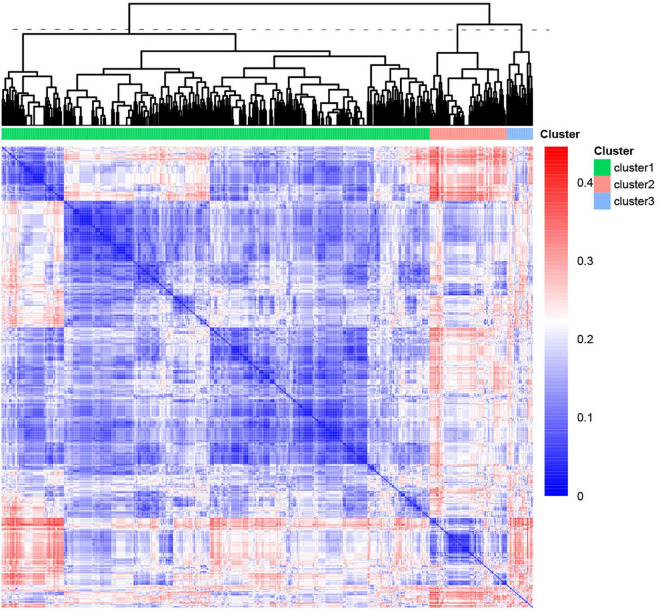
Heatmap of the phenotypic similarity clustering of PA patients. The heatmap was calculated by unsupervised clustering from the distance matrix of the phenotypic similarity of all patients. The line of dashes represented the height to cut the tree into three clusters. The color indicated the similarity between patients.

We performed unsupervised hierarchical clustering of the HPO database to obtain the different and similar phenotypes of clusters. The patients were classified into three clusters with numbers of 522, 94, and 32 (Figure 3). We analyzed the phenotypic distribution of the three clusters and found that a large number of HPO terms presented significantly different between three clusters, including TOF, VSD, SV, RVH, HRH, ASD, PDA, PFO, CA, ACA, AAC, tricuspid disease, and LSVC (Table 1). According to significant differences in HPO term distribution, we defined cluster 1 as being associated with TOF, VSD, RVH, and ACA and cluster 2 as being associated with HRH, ASD and tricuspid disease as the main morphological abnormalities. Cluster 3 presented higher frequency of SV, dextrocardia, and CA, but lower frequency of PDA and PFO.

### Clinical outcomes

**FIGURE 4:**
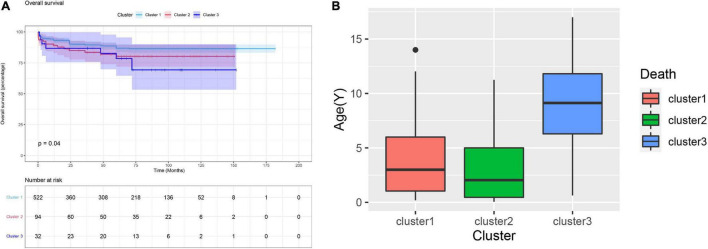
**(A)** Kaplan-Meier curve for clinical outcomes of three clusters. The Kaplan-Meier curve indicated the survival rate of cluster 1 was significant higher than cluster 2 and 3 (*p* = 0.04). **(B)** The age of death of the patients in cluster 3 was significantly higher than that of the cluster 1 and 2.

During the median of 5.3 years of follow-up, 85 (12.8%) patients suffered all-cause mortality. The overall survival rate at 5 years was 87.2%, and the three clusters were 88.7, 80.3, and 78.5%, respectively. Kaplan-Meier analysis revealed that the mortality rate in cluster 1 was significantly lower than the rates in cluster 2 and 3 (*p* = 0.04, Figure 4A). As for the age of death, the cluster 3 was significantly higher than the cluster 1 and 2, which might be related to the older surgical age (*p* = 0.003, Figure 4B).

In our cohort, the cluster, SV, abnormal spatial orientation of the cardiac segments (ASOCS), CA, atrioventricular canal defect (ACD), AAC, abnormal atrioventricular valve morphology (AAVM), mitral atresia (MA), and LSVC were risk factors for PA patients. Then, multivariable analysis revealed that AAC (3.737, *p* = 0.011) and LSVC (2.308, *p* = 0.003) were associated with an increased risk of mortality (Table 2). The univariate Cox proportional regression analysis of selected factors for mortality is presented in Table 3.

**TABLE 2:** Multivariable Cox proportional hazards regression analysis of death.

Variables	Hazard ratio	*P*-value
Cluster (1)	0.754 (0.304–1.871)	0.543
Cluster (2)	0.725 (0.235–2.241)	0.577
Age	0.967 (0.897–1.042)	0.378
Female	1.337 (0.849–2.105)	0.21
TOF	0.55 (0.267–1.134)	0.105
VSD	0.701 (0.313–1.571)	0.388
ASD	0.891 (0.543–1.463)	0.648
RVH	1.239 (0.752–2.039)	0.4
ACA	1.029 (0.625–1.693)	0.911
HRH	1.283 (0.486–3.385)	0.615
SV	1.135 (0.448–2.877)	0.79
Dextrocardia	1.145 (0.508–2.584)	0.744
CA	1.61 (0.539–4.815)	0.394
LSVC	2.308 (1.338–3.98)	0.003*
TR/TI	1.157 (0.545–2.459)	0.704
MA	2.233 (0.366–13.633)	0.384
AAC	3.737 (1.361–10.263)	0.011*
ASOCS	2.208 (0.814–5.053)	0.129
ACD/ECD	1.391 (0.414–4.679)	0.594
AAVM	1.752 (0.586–5.242)	0.316

*P-value < 0.05 was considered statistically significant. TOF, tetralogy of Fallot; VSD, ventricular septal defect; ASD, atrial septal defect; RVH, right ventricular hypertrophy; ACA, aortopulmonary collateral arteries; HRH, hypoplastic right heart; SV, single ventricle; CA, common atrium; LSVC, persistent left superior vena cava; TR/TI, tricuspid regurgitation/tricuspid insufficiency; MA, mitral atresia; AAC, abnormal atrioventricular connection; ASOCS, abnormal spatial orientation of the cardiac segments; ACD/ECD, atrioventricular canal defect/Endocardial cushion defect; AAVM, abnormal atrioventricular valve morphology.

**TABLE 3:** Univariable Cox proportional hazards regression analysis of death.

Variables	Hazard ratio	*P*-value
Cluster	1.513 (1.093–2.094)	0.02*
Female	1.359 (0.883–2.092)	0.162
Age at definitive surgery	0.972 (0.906–1.043)	0.418
History	0.922 (0.128–6.626)	0.935
Surgical strategy	1.112 (0.847–1.458)	0.448
BMI	1.004 (0.955–1.056)	0.874
LVEF	0.983 (0.967–1.000)	0.079
TOF	0.492 (0.246–0.983)	0.028*
VSD	0.533 (0.335–0.847)	0.011*
ASD	1.378 (0.89–2.135)	0.155
PDA	1.186 (0.704–1.999)	0.516
PFO	0.748 (0.459–1.217)	0.232
RVH	1.066 (0.674–1.688)	0.785
ACA	0.888 (0.573–1.375)	0.593
HRH	1.14 (0.497–2.617)	0.761
SV	2.841 (1.571–5.14)	0.002*
Dextrocardia	1.966 (1.015–3.808)	0.066
CA	3.932 (1.966–7.867)	0.001*
LSVC	2.179 (1.328–3.574)	0.004*
PAS	0.733 (0.297–1.81)	0.48
RAA	1.106 (0.51–2.399)	0.801
TR/TI	1.106 (0.622–1.964)	0.735
TS	0.384 (0.053–2.761)	0.261
TA	0.366 (0.051–2.63)	0.234
DTV	2.094 (0.767–5.719)	0.195
MA	6.772 (1.661–27.608)	0.04*
HLH	1.476 (0.205–10.607)	0.716
LVH	2.521 (0.351–18.114)	0.424
AAC	4.477 (2.057–9.746)	0.002*
ASOCS	3.681 (1.842–7.356)	0.002*
ACD/ECD	3.149 (1.45–6.835)	0.013*
AAVM	4.133 (1.9–8.987)	0.003*

*P-value < 0.05 was considered statistically significant. LVEF, left ventricular ejection fraction; TOF, tetralogy of Fallot; VSD, ventricular septal defect; ASD, atrial septal defect; PDA, patent ductus arteriosus; POF, patent foramen ovale; RVH, right ventricular hypertrophy; ACA, aortopulmonary collateral arteries; HRH, hypoplastic right heart; SV, single ventricle; CA, common atrium; LSVC, persistent left superior vena cava; PAS, pulmonary artery stenosis; RAA, right aortic arch; TR/TI, tricuspid regurgitation/tricuspid insufficiency; DTV, dysplastic tricuspid valve; TS, tricuspid stenosis; TA, tricuspid atresia; MA, mitral atresia; HLH, hypoplastic left heart; LVH, Left ventricular hypertrophy; AAC, abnormal atrioventricular connection; ASOCS, abnormal spatial orientation of the cardiac segments; ACD/ECD, atrioventricular canal defect/Endocardial cushion defect; AAVM, abnormal atrioventricular valve morphology.

## Discussion

Patients with PA are a heterogeneous population in terms of underlying anatomy and pathology (2). Heterogeneous phenotypes could lead to different surgical decisions and prognoses. However, the clinical classification of PA remains vague with different versions having been proposed (15, 16). Herein, we aim to explore and confirm the risk stratification of HPO terms in complex congenital heart disease. In our study, we retrieved the HPO database and used unsupervised hierarchical clustering to classify all patients into 3 clusters with similar phenotypes.

Each cluster had specific phenotypic characteristics that were significantly different from the other clusters. The HPO terms TOF, VSD, RVH, and ACA were frequently in cluster 1. According to the survival analysis, cluster 1 presented a significantly lower all-cause mortality than clusters 2 and 3 (Figure 4A, *p* = 0.04). We hypothesized that these four phenotypes are relatively less harmful abnormal cardiac structures in patients with PA. Montanaro et al. reported that patients with VSD had a better prognosis than those with pulmonary atresia with an intact ventricular septum (PA-IVS) (2). In such cases, it is usually true that blood flows through the ventricular septal defect into the left heart and the ductus arteriosus as the channel of flow into confluent pulmonary arteries. In general, the pulmonary vasculature in PA-VSD is reasonably well developed. However, patients with PA-IVS created a complete physical separation between the right ventricle and the pulmonary arteries, and the blood flow of these patients could only enter the left heart through the foramen ovale. It is manifested by less blood flow to the pulmonary artery, so pathology is characterized by severe hypoplasia or absence of the central pulmonary arteries and, in general, is associated with the presence of multiple aortopulmonary collaterals. In the meantime, PA-IVS is driven primarily by varying degrees of right ventricle and tricuspid valve hypoplasia, which exacerbates the severity of disease and leads to lower patient survival than PA-VSD (17–20). In summary, patients with PA-VSD have a better prognosis than those with PA-IVS. To study the haemodynamics of PA-VSD and PA-IVS, pulmonary atresia with VSD is similar to another condition called TOF, with the exception that the infundibulum either narrows to a blind end point or terminates at an imperforate pulmonary valve plate (17). In the study of Sharma et al., PA with VSD is an extreme form of TOF with characteristic right ventricular hypertrophy (21). It is well known that right ventricular hypertrophy is one of the four main manifestations of TOF (17). In conclusion, we believe that the presence of TOF, VSD, and RVH is concomitant. In infants with PA, the central pulmonary arteries may be hypoplastic, discontinuous, or absent, and the pulmonary vascular bed may be supplied with blood flow from aortopulmonary collaterals (22). Therefore, patients with aortopulmonary collaterals had a more mature pulmonary artery and a higher postoperative survival rate. Therefore, we consider them as an overall phenotype and relatively mild heart malformations in patients with PA.

Cluster 2 was mainly associated with dysplasia of the right heart, including HRH, ASD, and tricuspid disease. It has been proven that PA-IVS is driven primarily by varying degrees of right ventricle and tricuspid valves hypoplasia (18, 19, 23). In our cohort, the incidence of abnormal tricuspid valve was significantly higher in cluster 2, such as tricuspid regurgitation, dysplastic tricuspid valve, tricuspid stenosis, and tricuspid atresia. This indicates that tricuspid disease is a serious developmental abnormality for PA patients. Salvin et al., demonstrated that fetal tricuspid valve size and growth are predictors of pulmonary atresia with intact interventricular septum outcome. Fetuses with better tricuspid valve development had a better prognosis (24), which is in part consistent with our results. HRH is another typical phenotype in cluster 2. Patients with pulmonary atresia who have hypoplastic right heart syndrome frequently have a residual right-to-left shunt at the atrial level, resulting from resistance to right ventricular inflow. This may be a result of an inadequate right ventricular volume, diminished right ventricular compliance, and abnormalities of the tricuspid valve (25). Therefore, complicating HRH and tricuspid are the causes of increased mortality in patients with PA. In a study of PA-IVS, Xiaomin He et al. described the presence of ASD and PFO in all patients (26). Chubb et al. and Wright et al. also documented that some PA-IVS patients required atrial septal defect repair (4, 23). These results suggest that ASD is more common in patients with PA-IVS, which is consistent with our results.

For cluster 3, we found that SV, Dextrocardia and CA were the dominant phenotypes. Obviously, patients in cluster 3 had significantly more serious heart malformations than those in the other two clusters. Infants with a single ventricle have a high risk of death during the early years of life and undergo Fontan (27, 28). In addition, dextrocardia is found in a significant proportion of patients with a single ventricle. The outcomes of a single ventricle seem to be worse in patients with dextrocardia than in those with laevocardia (29, 30). These factors could partly explain why the patients in cluster 3 had a much higher mortality than those in clusters 1 and 2. Moreover, the age at definitive surgery in this cluster was significantly older than that in the other clusters. Zheng et al. reported that younger patients had a better opportunity for initial intervention. Because their RV still had adequate growth potential, the primary intervention was generally offered to promote RV growth (26). This is one of the reasons for the poorer prognosis in cluster 3.

According to these results and our previous findings (11, 14), we believe that the HPO database is a powerful tool for phenotypically based risk stratification of complex congenital heart diseases, and may even be extended to other systemic developmental defects and heterogeneous diseases. We used this method cleverly to divide patients into three clusters and successfully identified high-risk phenotypes. Based on phenotypes and all-cause mortality in all three clusters, we found that PA patients with VSD and aortopulmonary collaterals had a better prognosis, while HRH, tricuspid valve disease, severe heart malformations (single ventricle, dextrocardia) and older surgical age were risk factors that increased mortality. The clinical phenotypes of newly admitted patients can be compared with the PA phenotypic database to find the cluster of patients with the closest phenotype. Thus, the prognosis of this newly admitted patient can be judged, which is better in cluster 1 and worse in cluster 2 or 3.

## Limitations

As this is a retrospective-cohort and observational study at a single medical center, selection bias is inevitable. The median follow-up time was just 5.3 years, and a longer follow-up may provide better clarification of clinical status and long-term sequelae after definitive surgery. In addition, we analyzed all-cause mortality, because not all exact causes of death could be obtained during the follow-up. A limitation of various subtypes and complex phenotypes was also noted in our study. Valvular (aortic valve, mitral, tricuspid) function, including regurgitation/insufficiency and stenosis, could be divided into mild, moderate and severe. However, the HPO terms did not include the levels of subdivision, so these subtypes were not calculated during the process of clustering. In addition, a few HPO terms in database could not be searched in the R package “ontologySimilarity,” including “Coronary-pulmonary artery fistula,” “Situs inversus with levocardia,” and “Aortopulmonary collateral arteries,” which might cause bias.

## Conclusion

Our study reported a large cohort with clinical phenotypic, surgical strategy and long time follow-up. In addition, we provided a precise classification and successfully risk stratification for patients with PA.

## Data availability statement

The raw data supporting the conclusions of this article will be made available by the authors, without undue reservation.

## Author contributions

BS: study design, data reduction, follow-up, statistical analysis, and article writing. HS: study design and article writing. XS: data reduction and follow-up. FL: follow-up. TL: data reduction. ZZ: study guidance. BS and ZZ: responsible for the overall content as guarantors. All authors contributed to the article and approved the submitted version.

## Footnotes

1
https://hpo.jax.org/app/

2
https://rdrr.io/cran/ontologySimilarity/f/vignettes/ontologySimilarity-examples.Rmd
