# Supplementary material for: Human phenotype ontology annotation and cluster analysis for pulmonary atresia to unravel clinical outcomes
Source: Front Cardiovasc Med. 2022 Jul 29;9:898289. doi: 10.3389/fcvm.2022.898289 (PMC9372274; doi:10.3389/fcvm.2022.898289)
Supplement: Supplementary file 1 [file Table_1.docx]

Supplementary Material

# Supplementary Table 1

Table S1. HPO terms in our cohort HPO.

| Term Identifier | Term Name | Abbreviation |
| --- | --- | --- |
| HP:0001636 | Tetralogy of Fallot | TOF |
| HP:0004415 | Pulmonary artery stenosis | PAS |
| HP:0004935 | Pulmonary artery atresia | PAA |
| HP:0010444 | Pulmonary insufficiency | PI |
| HP:0001629 | Ventricular septal defect | VSD |
| HP:0001631 | Atrial septal defect | ASD |
| HP:0005120 | Abnormal cardiac atrium morphology | ACAM1 |
| HP:0030718 | Right atrial enlargement | RAE |
| HP:0011565 | Common atrium | CA |
| HP:0001655 | Patent foramen ovale | PFO |
| HP:0001643 | Patent ductus arteriosus | PDA |
| HP:0001719 | Double outlet right ventricle | DORV |
| HP:0001705 | Right ventricular outlet tract obstruction | RVOTO |
| HP:0001640 | Cardiomegaly | Cardiomegaly |
| HP:0001667 | Right ventricular hypertrophy | RVH |
| HP:0005133 | Right ventricular dilatation | RVD |
| HP:0010954 | Hypoplastic right heart | HRH |
| HP:0001651 | Dextrocardia | Dextrocardia |
| HP:0011599 | Mesocardia | Mesocardia |
| HP:0001750 | Single ventricle | SV |
| HP:0004383 | Hypoplastic left heart | HLH |
| HP:0001712 | Left ventricular hypertrophy | LVH |
| HP:0005180 | Tricuspid regurgitation/ Tricuspid insufficiency | TR/TI |
| HP:0010446 | Tricuspid stenosis | TS |
| HP:0011662 | Tricuspid atresia | TA |
| HP:0001702 | Abnormal tricuspid valve morphology | ATVM |
| HP:0030732 | Dysplastic tricuspid valve | DTV |
| HP:0010316 | Ebstein anomaly of the tricuspid valve | EATV |
| HP:0001659 | Aortic regurgitation/Aortic insufficiency | AR/AI |
| HP:0001650 | Aortic valve stenosis | AVS |
| HP:0001647 | Bicuspid aortic valve | BAV |
| HP:0001653 | Mitral regurgitation/Mitral insufficiency | MR/MI |
| HP:0011560 | Mitral atresia | MA |
| HP:0001633 | Abnormal mitral valve morphology | AMVM |
| HP:0006695 | Atrioventricular canal defect/Endocardial cushion defect | ACD/ECD |
| HP:0006705 | Abnormal atrioventricular valve morphology | AAVM |
| HP:0011546 | Abnormal atrioventricular connection | AAC |
| HP:0001669 | Transposition of the great arteries | TGA |
| HP:0006704 | Abnormal coronary artery morphology | ACAM2 |
| HP:0002092 | Pulmonary arterial hypertension | PAH1 |
| HP:0004414 | Abnormality of the pulmonary artery | APA |
| HP:0004971 | Pulmonary artery hypoplasia | PAH2 |
| HP:0011718 | Abnormality of the pulmonary veins | APV |
| HP:0005301 | Persistent left superior vena cava | LSVC |
| HP:0011534 | Abnormal spatial orientation of the cardiac segments | ASOCS |
| HP:0011590 | Double aortic arch | DAA |
| HP:0001679 | Abnormal aortic morphology | AAM |
| HP:0012020 | Right aortic arch | RAA |
| HP:0005135 | Abnormal T-wave | ATW |
| HP:0012249 | Abnormal ST segment | ASTS |
| HP:0011712 | Right bundle branch block | RBBB |
| HP:0006682 | Premature ventricular contraction/Ventricular premature beat | PVC/VPB |
